# Supplementary figures and images for: The Transcription Factor Nfix Requires RhoA-ROCK1 Dependent Phagocytosis to Mediate Macrophage Skewing during Skeletal Muscle Regeneration
Source: Cells. 2020 Mar 13;9(3):708. doi: 10.3390/cells9030708 (PMC7140652; doi:10.3390/cells9030708)

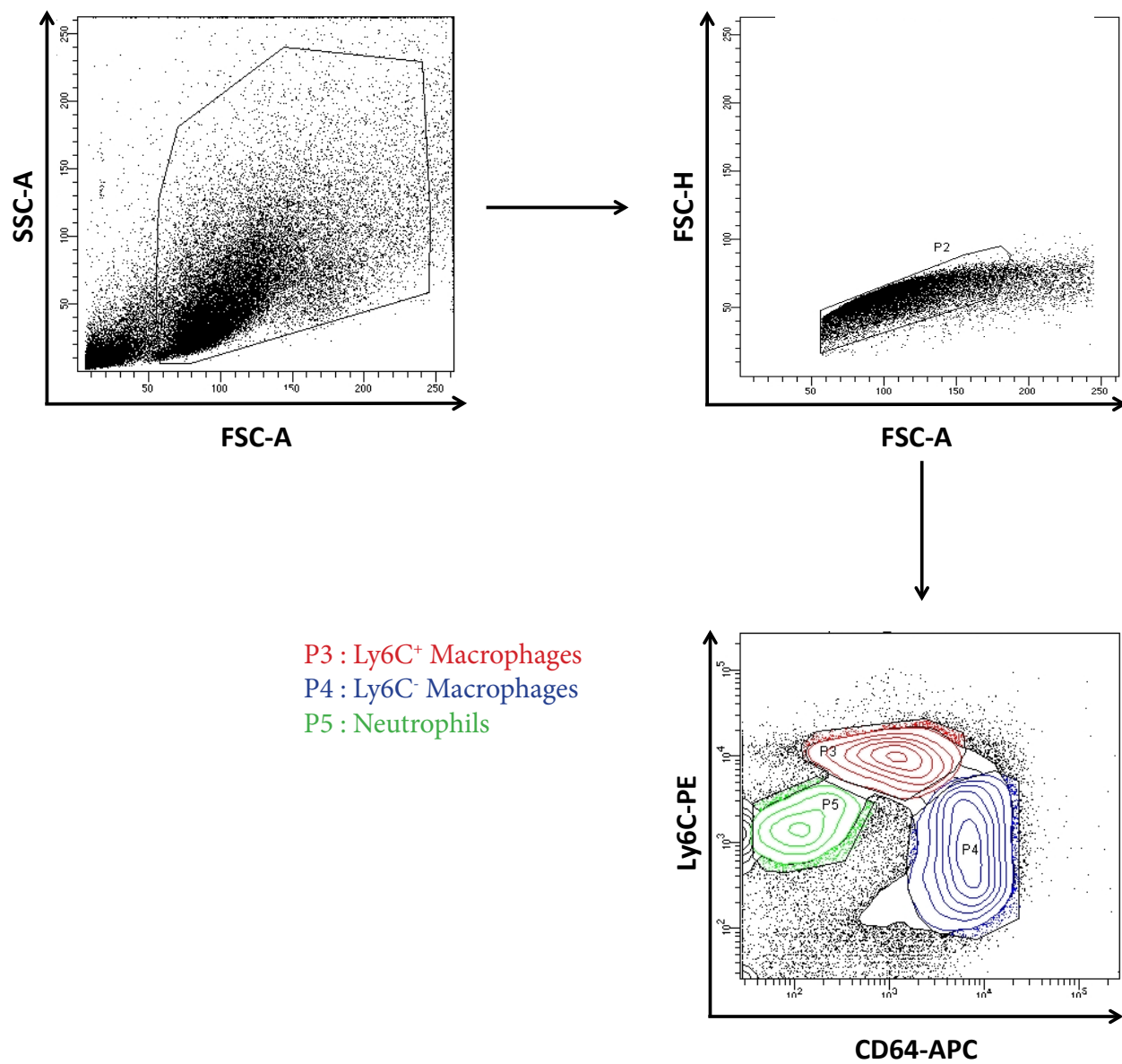

Figure S1

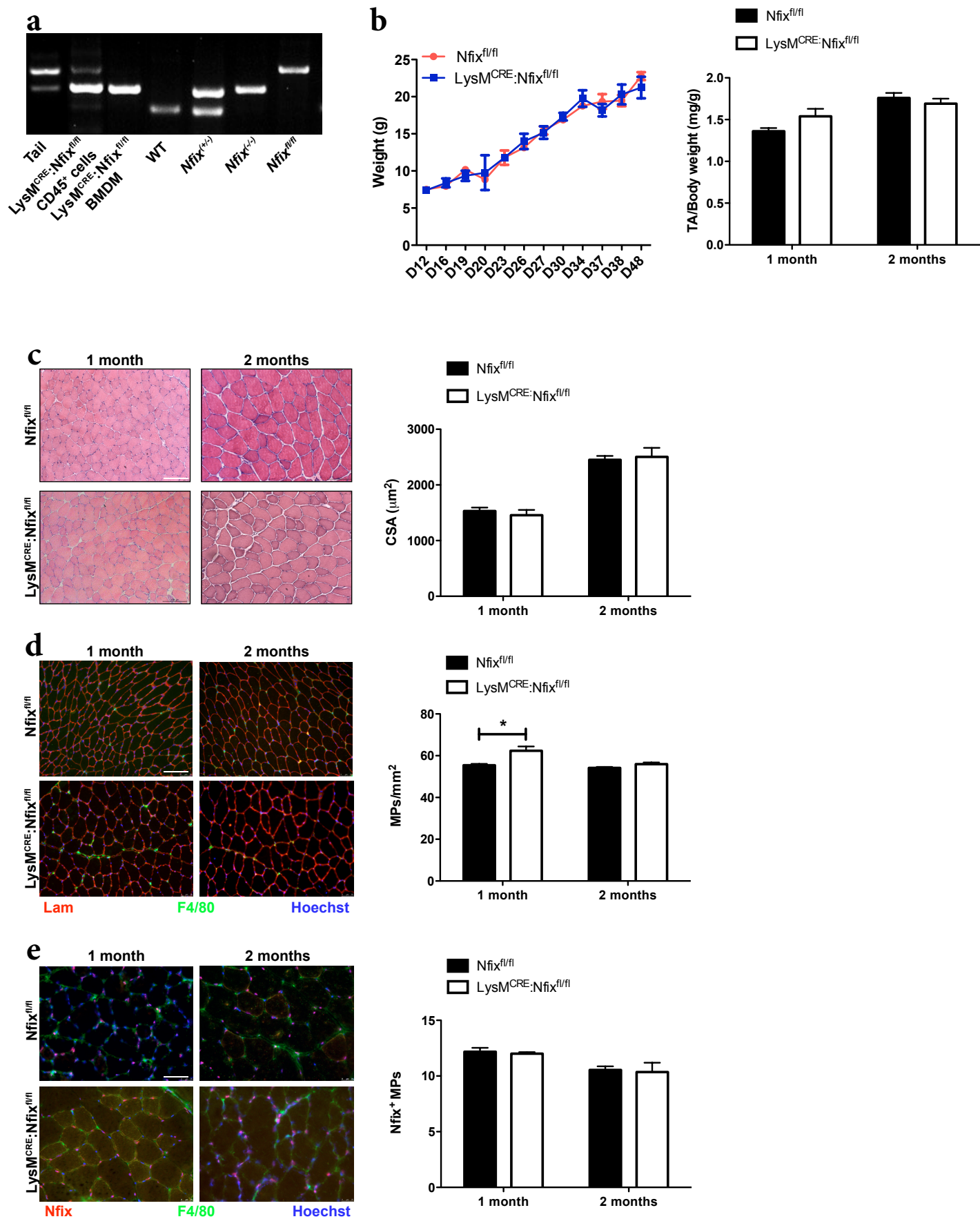

Figure S2

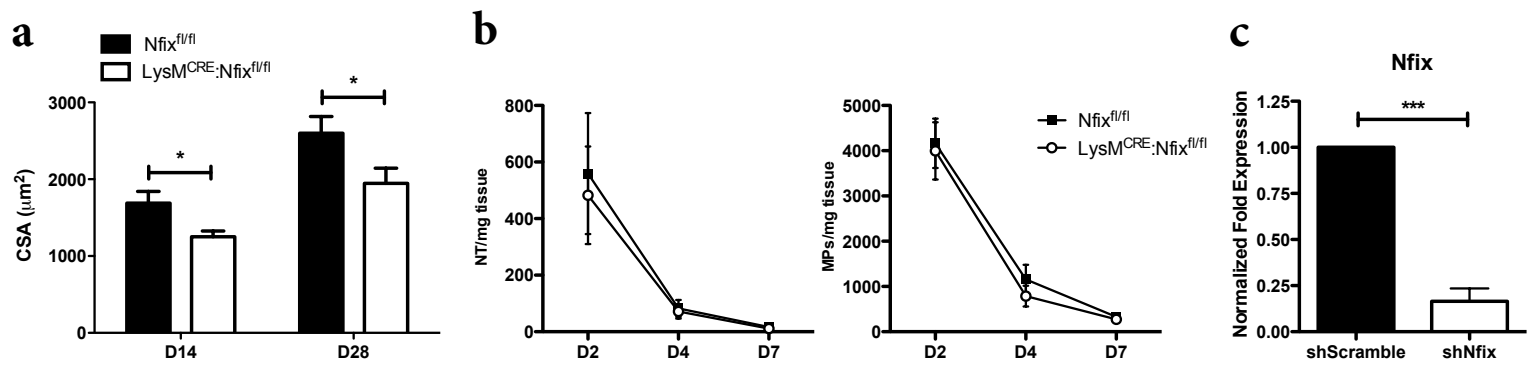

Figure S3

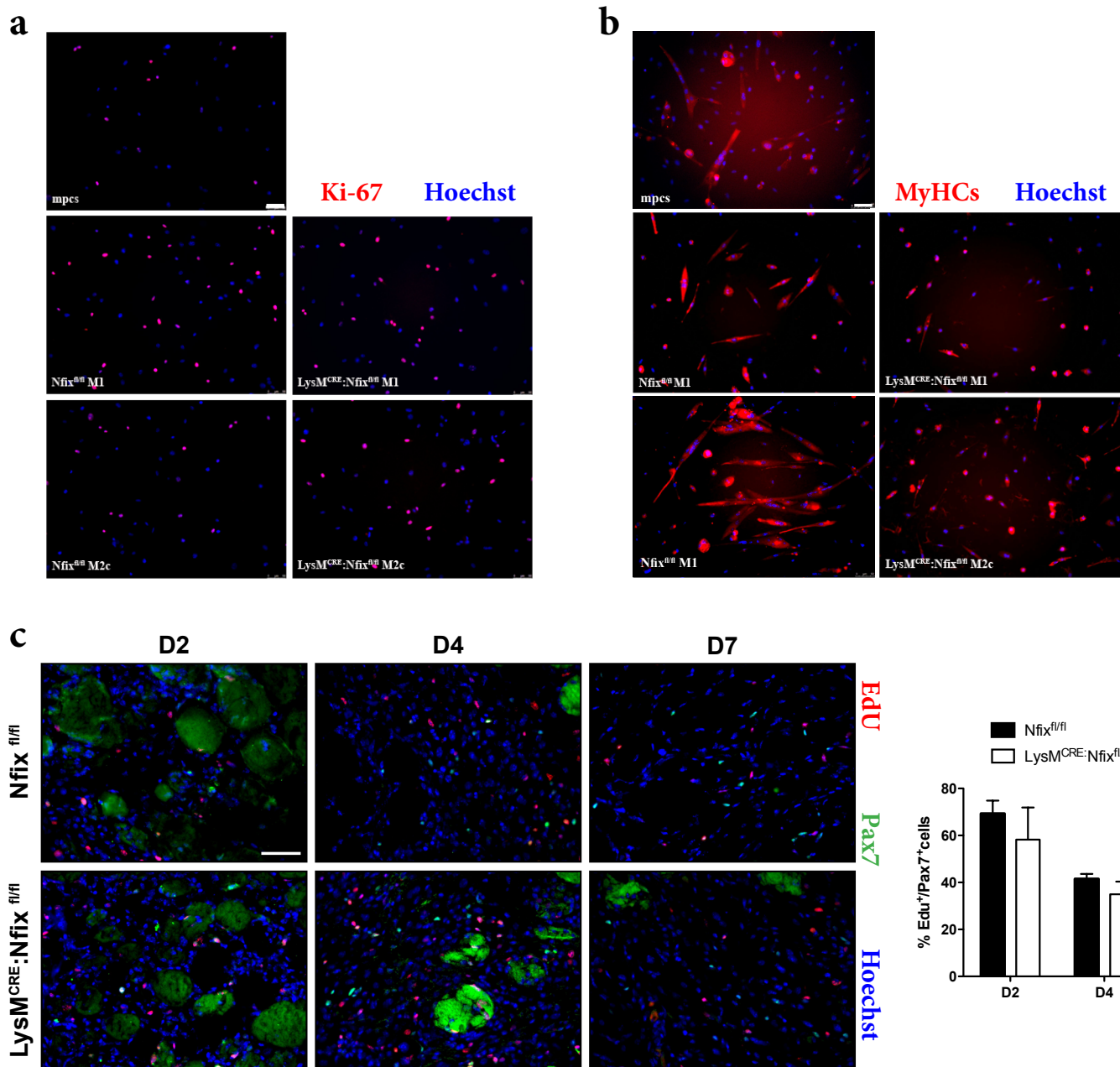

Figure S4

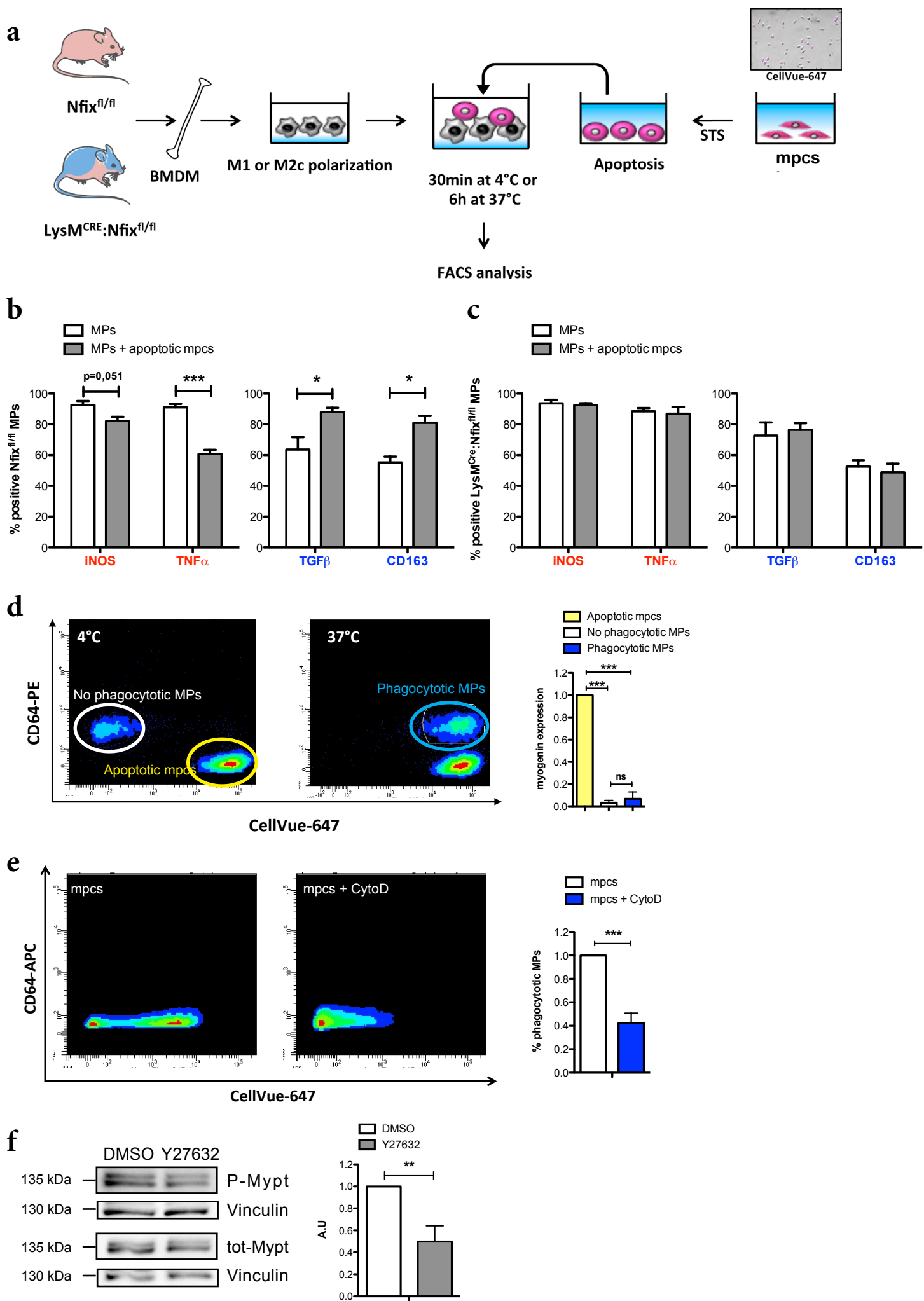

**Figure S5**

Supplement: Supplementary file 1 [file cells-09-00708-s001.pdf]
